# Resolution of long-standing persistent atrial fibrillation after coronary revascularization: a case report on this rare reversible cause of atrial fibrillation

**DOI:** 10.1093/ehjcr/ytag084

**Published:** 2026-02-04

**Authors:** Suresh Kumar Sukumaran, Rahul Aziz Seena, Sasinthar Rangasamy, Sathish Kumar Subbaraj, Kannan BRJ

**Affiliations:** Department of Cardiology, Vadamalayan Hospitals, Madurai 625002, India; Department of Cardiology, Vadamalayan Hospitals, Madurai 625002, India; Department of Cardiology, Vadamalayan Hospitals, Madurai 625002, India; Department of Cardiology, Vadamalayan Hospitals, Madurai 625002, India; Department of Cardiology, Vadamalayan Hospitals, Madurai 625002, India

**Keywords:** Tachycardiacdot, Atrial fibrillationcdot, Percutaneous transluminal coronary angioplastycdot, Myocardial perfusioncdot Reversible atrial fibrillation, Cardiac Positron Emission Tomography, Cardiac Computed Tomography, Case Report

## Abstract

**Background:**

Atrial fibrillation (AF) commonly coexists with coronary artery disease (CAD) due to overlapping risk factors. Although ischaemia can promote atrial arrhythmogenesis, sustained restoration of sinus rhythm after revascularization alone is unusual.

**Case summary:**

A 55-year-old man with hypertension and long-standing persistent AF (three years) was referred for pulmonary vein isolation. Echocardiography showed mild LV dysfunction (LVEF 48%) and basal septal thinning. Rest 99mTc-sestamibi myocardial perfusion SPECT (MPS) demonstrated moderate apical and mild to moderate inferior wall defects; 18F-FDG PET/CT with myocardial suppression showed no focal myocardial FDG uptake to suggest active inflammation. Coronary angiography demonstrated critical proximal LAD stenosis (95%) and minor RCA disease (30%). Following LAD PCI (3 mm × 15 mm DES), the patient spontaneously converted to sinus rhythm within two hours and has remained in sinus rhythm for 6 months without antiarrhythmic drugs. LV function normalized at 1 month.

**Discussion:**

AF usually arises from pulmonary-vein triggers acting on a remodelled atrial substrate; chronic comorbidities (hypertension, obesity, diabetes, sleep apnoea), accelerated fibrosis, conduction slowing, and re-entry. CAD is prevalent in AF cohorts (17–46.5%), reflecting shared risk profiles and potential ischaemic effects on the atria. Experimental work shows atrial ischaemia shortens refractoriness, increases dispersion of repolarization, and heightens AF inducibility. Coronary occlusion (particularly RCA) facilitates both triggers and substrate. Clinically, reports conflict on whether coronary artery disease worsens post-ablation outcomes: large registries found no independent association between CAD burden and AF recurrence, while other studies observed higher recurrence in CAD with benefit from revascularization prior to ablation. Our case adds a rare but persuasive datapoint: ischaemia-driven AF that terminated immediately after PCI without ablation or antiarrhythmics, followed by durable sinus rhythm and rapid left ventricular recovery—features that argue causality.

**Conclusion:**

In atrial fibrillation patients with coexisting CAD, ischaemia may be a reversible driver of arrhythmia. This case demonstrates immediate and sustained restoration of sinus rhythm after PCI without antiarrhythmics or ablation. Incorporating ischaemia evaluation into pre-ablation assessment may avert invasive procedures and optimize outcomes in selected individuals.

Learning points

- Ischaemia can be a reversible driver of atrial fibrillation in selected patients.
- Systematic ischaemia evaluation may be considered in selected patients before ablation in AF where clinical suspicion of coronary artery disease exists.
- Revascularization may restore sinus rhythm and improve LV function when AF is ischaemia-mediated.

## Introduction

AF is the most common sustained arrhythmia, with major morbidity from stroke and heart failure.^1^ Mechanistically, AF reflects an interaction of focal triggers and atrial substrate remodelling under autonomic influence.^1–3^ Coronary artery disease (CAD) commonly coexists with AF due to shared risk factors,^2–4^ and observational studies report CAD in 17–46.5% of AF patients.^4–7^ While ischaemia can promote atrial electrical instability,^8^ durable restoration of sinus rhythm after coronary revascularization without ablation is rarely documented. We present a case of long-standing persistent AF reverting to sinus rhythm within hours of PCI, highlighting ischaemia as a potentially reversible cause.

## Summary figure

**Figure:**
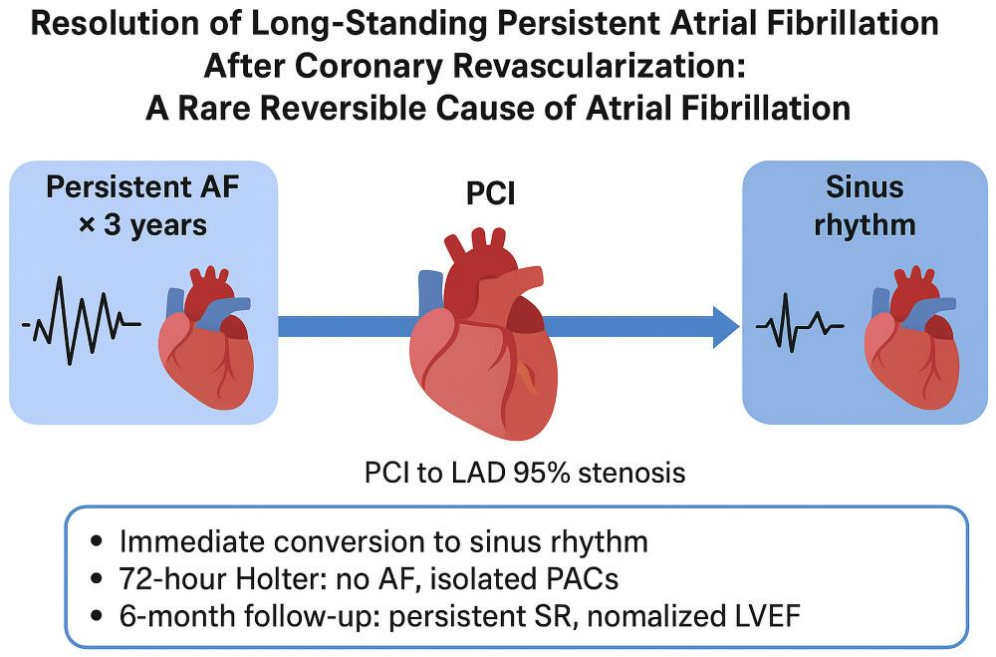


## Case presentation

A 55-year-old man with hypertension and persistent AF for three years (initially detected during pre-operative assessment) was referred for pulmonary vein isolation after pharmacologic cardioversion with amiodarone had failed. He was currently taking metoprolol 25 mg once daily, reported dyspnoea only at maximal exertion, and had no other limiting symptoms.

**Figure 1:**
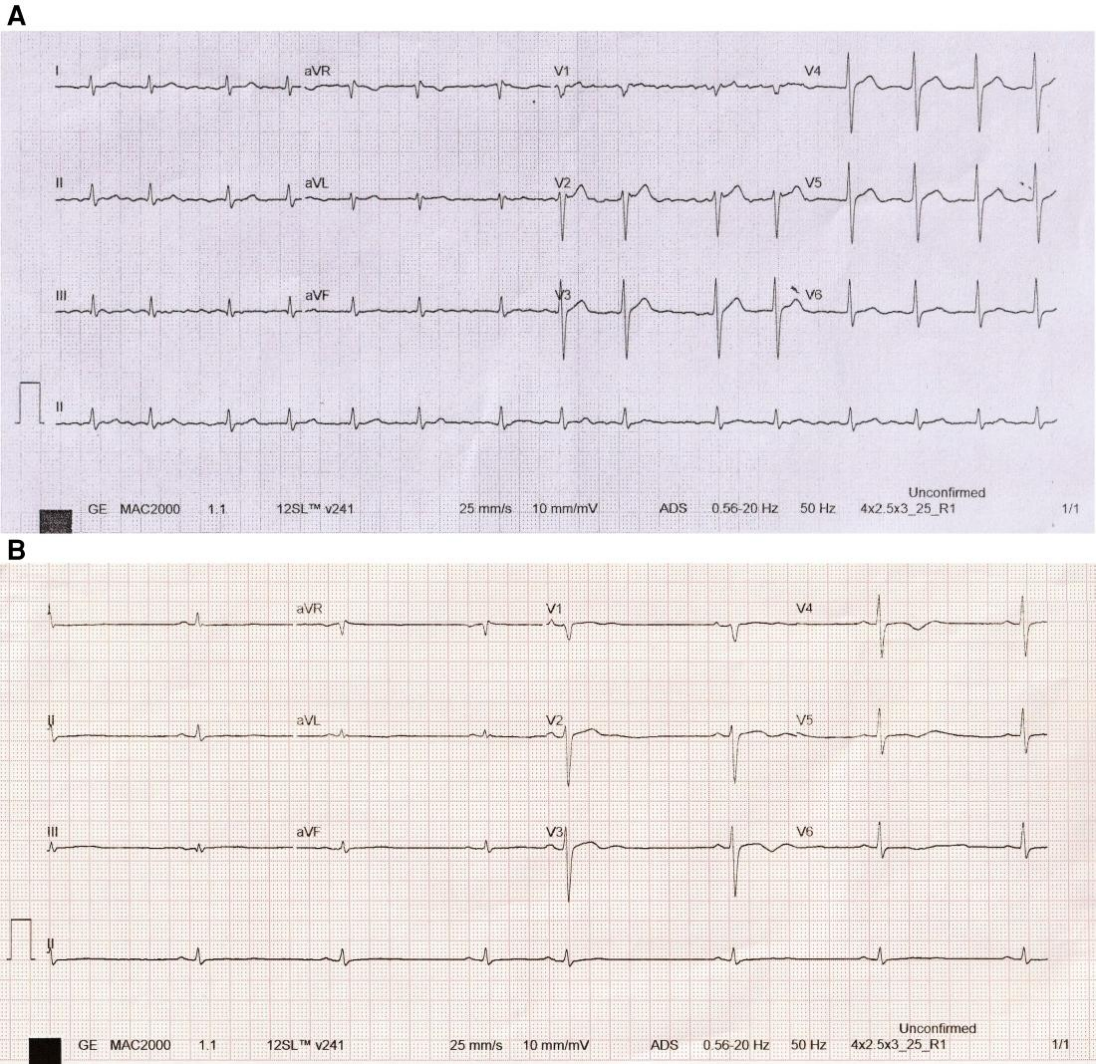
Electrocardiogram. (*A*) Preoperative electrocardiogram demonstrates fine atrial fibrillation at approximately 90 bpm, with a normal QRS axis and duration, and without significant ST–T segment abnormalities. (*B*) Immediate postoperative electrocardiogram shows restoration of sinus rhythm at around 48 bpm, with occasional premature atrial contractions.

Examination revealed irregular rhythm; ECG showed atrial fibrillation with controlled ventricular rate (*Figure 1A*). Echocardiography showed mild global LV hypokinesia (LVEF 48%) and basal septal thinning (6.9 mm). Initially, he was planned for elective cardioversion, but concern for cardiac sarcoidosis prompted further advanced imaging, including Myocardial Perfusion Scintigraphy (MPS) and 18F-FDG PET scan before elective cardioversion.

The myocardial suppression two-day protocol was used. 99mTc-sestamibi 8 mCi (≈296 MBq) was administered at rest with imaging at 60 min. For FDG PET, 18F-FDG 5.2 mCi (≈192 MBq) was injected after a 24-hour high-fat, very-low-carbohydrate diet and a 17-hour fasting period. Unfractionated heparin 4050 IU (≈50 IU/kg) was given 20 min before FDG, and fasting blood glucose was 105 mg/dL (≈5.8 mmol/L).^9^

**Figure 2:**
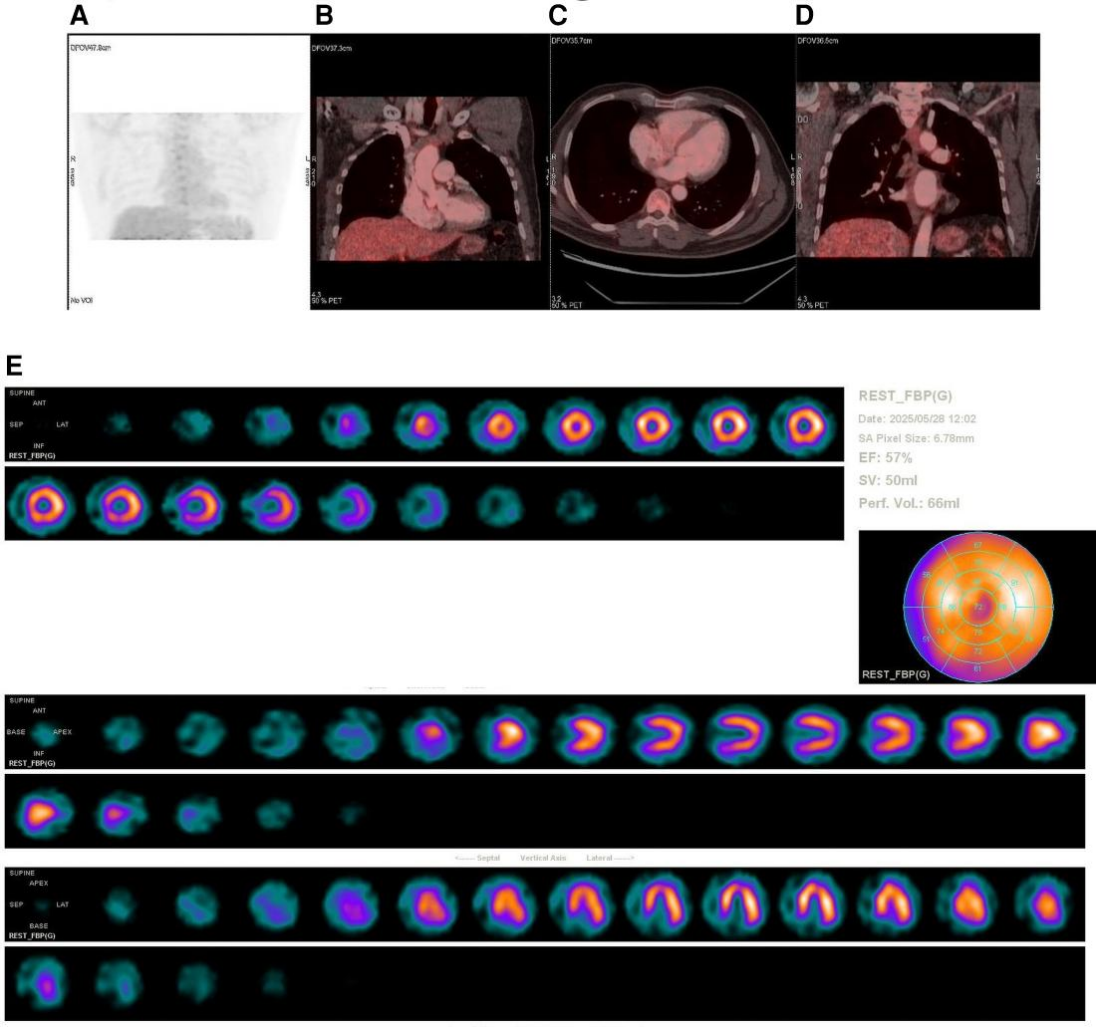
Nuclear medicine imaging. (*A*) Maximum-intensity projection (MIP) of cardiac 18F-FDG PET/CT after a myocardial-suppression protocol shows no focal or diffuse myocardial FDG uptake. (*B–C*) Fused PET/CT coronal and axial images with the heart in the field of view demonstrate physiologic myocardial FDG suppression. (*D*) Fused PET/CT coronal image focused on the mediastinum shows no FDG-avid lymphadenopathy. (*E*) Rest 99mTc-sestamibi myocardial perfusion scintigraphy (MPS): short-axis (SA), polar map, vertical long-axis (VLA), and horizontal long-axis (HLA) views demonstrate a moderate apical perfusion defect with mild–moderate mid- and basal-inferior defects.

Rest MPS demonstrated a moderate apical perfusion defect and mild to moderate mid—and basal-inferior wall defects. Limited cardiac 18F-FDG PET performed under a myocardial suppression protocol showed no focal or diffuse myocardial FDG uptake, arguing against active inflammatory/infective aetiology (*Figure 2*).^9,10^ Given the LAD-territory location of the apical abnormality and the equivocal nature of the inferior defects (where diaphragmatic attenuation is common), the nuclear medicine imaging impression favoured ischaemia —warranting invasive coronary evaluation.^11^

**Figure 3:**
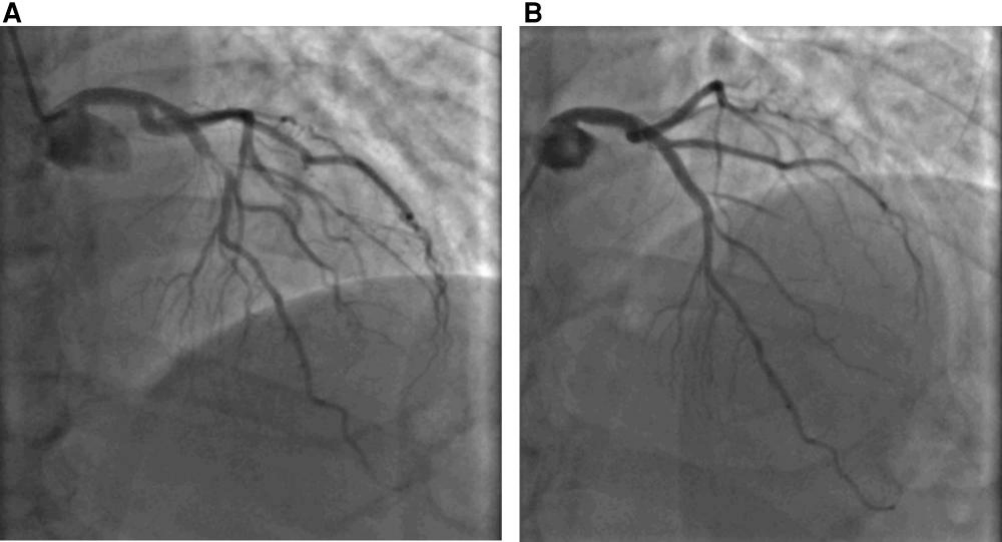
Coronary angiogram. (*A*) AP cranial coronary angiographic view demonstrating a critical 95% stenosis in the proximal left anterior descending artery. (*B*) AP cranial coronary angiographic view following deployment of a 3 mm × 15 mm stent across the proximal left anterior descending artery stenosis.

Hence, coronary angiography was done first before elective cardioversion to avoid any potential complications, and that revealed a tight proximal LAD stenosis (95%) (*Figure 3A*) and a minor proximal RCA lesion (30%). The patient underwent PCI with implantation of a 3 × 15 mm drug-eluting stent in the LAD (*Figure 3B*). Two hours post-PCI, he spontaneously converted to sinus rhythm (*Figure 1B*). A 72-hour Holter recording in the immediate postoperative period revealed no atrial fibrillation, with only isolated premature atrial contractions (<0.1%). Over 6 months of follow-up, he has remained in sinus rhythm without antiarrhythmics, and the LV systolic function normalized within 1 month, and he is asymptomatic.

## Discussion

Fundamentals of AF pathophysiology emphasize pulmonary-vein triggers operating on an atrial substrate shaped by fibrosis, dilatation, and metabolic/inflammatory stressors.^1–3^ Chronic hypertension and other cardiometabolic risks accelerate adverse remodelling, making long-standing persistent AF particularly resistant to spontaneous conversion; definitive rhythm control typically requires ablation or antiarrhythmic drugs.^1,5^

CAD is common among AF patients.^4–7^ In the AFFIRM cohort and other epidemiologic datasets, coexistence reflects shared risks and possibly direct ischaemic effects on atrial tissue.^4–6^ The atrial blood supply derives from the sinoatrial nodal/atrial branches—arising from the RCA in around 50–60% and the LCX in 40–50%—creating susceptibility to supply-demand mismatch and micro-ischaemia.^6^ Experimental studies elucidate mechanisms: Short-term atrial ischaemia via ionic/acid–base shifts (Na⁺/H⁺ exchange) shortens atrial refractoriness and fosters heterogeneity^12^; chronic coronary occlusion models increase AF triggers and maintenance.^13^

99mTc-sestamibi is a lipophilic cation that localizes in viable myocytes proportionally to blood flow and mitochondrial density, with minimal redistribution.^14^ On rest-only imaging, perfusion defects may reflect infarction/scar, prior ischaemic injury, or artefact; without attenuation correction or stress comparison, inferior wall artefact (e.g. diaphragmatic attenuation) and apical thinning cannot be excluded.^11,14^ 18F-FDG PET detects glucose-avid myocardial inflammation when physiological myocardial uptake is suppressed by dietary manipulation and fasting. Typical interpretive patterns include: (i) focal or focal-on-diffuse FDG uptake (± reduced perfusion) suggesting active inflammation; (ii) regions with reduced perfusion and absent FDG uptake suggesting scar; and (iii) reduced perfusion with preserved FDG uptake indicating viable hibernating myocardium.^9,10^ Rest perfusion defects on MPS together with absent myocardial FDG uptake argued against active inflammatory cardiomyopathy and, in the clinical context, suggested that ischaemia was also a contributor to both AF persistence and LV dysfunction—an inference supported by immediate post-PCI sinus rhythm restoration and rapid LVEF recovery.^9–11,14,15^

Clinical data on CAD’s impact on AF rhythm-control outcomes are mixed. The Leipzig registry (1310 patients) found no independent relationship between CAD presence/extent and post-ablation recurrence,^16^ and coronary stenosis location/severity did not predict outcomes in another imaging-guided study.^17^ Conversely, Hiraya *et al*. observed higher AF recurrence in CAD vs. non-CAD, with lower recurrence when PCI preceded PVI.^18^ Chen *et al*. similarly reported that obstructive CAD predicted poorer ablation success, while revascularization was associated with improved AF-free survival.^7^ Percutaneous transluminal coronary angioplasty may restore sinus rhythm in atrial fibrillation associated with acute coronary syndromes affecting the right or left circumflex coronary arteries.^19–21^ These divergent findings likely reflect heterogeneity in CAD severity, ischaemia burden, and whether revascularization addressed the active ischaemic driver of AF.

Our case is instructive in several respects. First, temporal proximity: conversion to sinus rhythm occurred within 2 hours of LAD revascularization, strongly suggesting ischaemia as the proximate mechanism rather than delayed remodelling.^7,12^ Second, durability: maintenance of sinus rhythm for 6 months without antiarrhythmics parallels observations that treating ischaemia can improve rhythm stability in selected CAD patients.^7,18^ Third, ventricular recovery: rapid LVEF normalization indicates reversible ischaemic cardiomyopathy rather than primary myopathic remodelling, aligning with an ischaemia-mediated atrial substrate or AF mediated cardiomyopathy.^12,13^ Finally, although atrial branches often originate from the RCA,^6^ LAD revascularization could still relieve atrial ischaemia via improved global perfusion, collateral flow, anatomical variability in atrial branch contribution, and reduced LV wall stress/left-atrial pressure, thereby stabilizing atrial electrophysiology.^12,13^

In contrast to cohorts showing no effect of CAD on ablation outcomes,^16,17^ this patient never required ablation; at least for the first 6 months, correcting the ischaemic supply–demand mismatch alone sufficed. Compared with series demonstrating benefit from PCI before ablation,^7,18^ our observation is an even stronger proof-of-principle that in a subset with ischaemia-driven AF, revascularization can be definitive. Pragmatically, these data support a work-up for occult ischaemia in AF patients with suggestive symptoms, LV dysfunction, perfusion defects, or high atherosclerotic risk, before committing to ablation.^4–7,16–18^

Certain constraints may affect the validity of this observation. Although the restoration of sinus rhythm occurred immediately after PCI, suggesting that myocardial ischaemia may have been a precipitating factor for AF, the contribution of rhythm restoration itself to LV function recovery cannot be excluded. AF-mediated cardiomyopathy may have also played a role in the initial LV dysfunction, and its reversal with sinus rhythm maintenance could have contributed to the observed improvement. Given the absence of a stress perfusion study, the relative impact of revascularization vs. rhythm normalization remains uncertain. While sinus rhythm was restored immediately following PCI and maintained during the 6-month follow-up, this temporal association alone does not establish causality. The possibility of spontaneous cardioversion cannot be excluded, and a longer follow-up period would be required to confirm durable rhythm stability.

## Conclusion

In atrial fibrillation patients with coexisting CAD, ischaemia may be a reversible driver of arrhythmia. This case demonstrates immediate and sustained restoration of sinus rhythm after PCI without antiarrhythmics or ablation. Incorporating ischaemia evaluation into pre-ablation assessment may avert invasive procedures and optimize outcomes in selected individuals.^4–7,16–18^

## Lead author biography


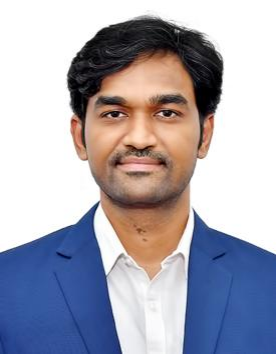


Dr. Suresh Kumar Sukumaran, MBBS, MD (General Medicine), DM (Cardiology), PDF (Cardiac Electrophysiology and Pacing), CCDS (IBHRE, USA), is a Consultant Cardiac Electrophysiologist and Interventional Cardiologist at Vadamalayan Hospitals and Postgraduate Institute of Medical Sciences, Madurai, Tamil Nadu, India. He completed his Fellowship in Cardiac Electrophysiology and Pacing (2022–2024) and DM in Cardiology (2019–2021) at JIPMER, Pondicherry, following his MD (2014–2017) and MBBS (2007–2013). Hosts a robust portfolio of research and clinical insights, evidenced across high-impact publications and advanced procedural expertise.

## Data Availability

The data that support the finding of this case report are available from the authors upon request.

## Patient perspective

The patient expressed surprise and relief that a single procedure resolved both his coronary narrowing and long-standing arrhythmia, avoiding ablation or long-term antiarrhythmic therapy.
